# Phrenology and Life Vocations

**Published:** 1903-08

**Authors:** E. G. Ferguson

**Affiliations:** Macon, Ga.


### PHRENOLOGY AND LIFE VOCATIONS.*

### TO ESTABLISH PERMANENT PHRENOLOGICAL EXAMINERS FOR ALL PUBLIC SCHOOL DISTRICTS, WHOSE DUTY IT SHALL BE TO EXAMINE PHRENOLOGICALLY ALL CHILDREN EACH SCHOOL TERM AND DETERMINE THE VOCATION IN LIFE THEY SHOULD FOLLOW.

BY E. G. FERGUSON, M.D.,

MACON, GA.

To my mind phrenology is almost as much of a science as mathematics, and would give guidance to the happy future of the child in matured years in its walk in life. Many parents want their child to be a professional man, for instance a lawyer, when his brain

*Read before the Macon Medical Society, May 5, 1903.

capacity is unequal to the proper discharge of the duties devolving upon him in the comprehension of the intricacies of the law. He studies law and makes a failure. Still, laboring under the impression that, as he has studied law, it is his profession, he continues to practice it. Whereas, if his poor little head had been examined by a competent phrenologist, he would have advised him to go to the butcher-pen and skin a cow instead of the unfortunate clients who fell into his legal net; or seek the plow-handles and cultivate corn. In either of these pursuits he would be contented and happy, as well as successful, while the little brain could never succeed at law. The same holds good in medicine. The misguided creature imagines he can study medicine, attain a certain proficiency and become a doctor. Having no capacity for the profundity of the ramifications of the respective sciences embraced in the study, he gets a smattering of the various branches, is graduated as an M.D. by some unscrupulous school, and turned loose on the world to practice the healing art. The fact of his having an M.D. to his name is a passport to the sick-room, where he is as competent to look after the pathological changes in that patient who has submitted his case to his charge as a bulldog is to rectify the disintegrations of a lock on a musket.

The competent mechanic in wood is confronted with a plug head who, after a fashion, has acquired a partial knowledge of woodwork through observation, and yet, deficient in knowledge of true structural construction, half-way meets the ends desired, and on the victim of his audacity leaves an impression of competency. Others go to the machine shops, thinking they can learn the art of mechanism by application for a number of years, and, step by step, after hard work, they acquire a certain amount of knowledge in handling tools, but never a knowledge of the first principles of mechanics, for it is not in them. The gift is not there, and in places a whitewash brush in their hands would be more creditable and better results be obtained. The question will be asked what is phrenology, that so much stress should be placed upon it. Take four skulls, one of an eminent divine, one of an atheist, one of a politician, and one of a murderer. Each and all are as distinct one from the other as daylight is from darkness.

The skull of the eminent divine is so formed that the most casual observer can at a glance see it differs from all others in its conformations. The faculty or bump of reverence is as patent to the sight as a mountain is on a clear day. The anterior or frontal portion is so far developed as to admit of no doubt. The atheist has no frontal development, but slopes back from the eyebrows to the back part of the head, which is largely developed, wherein all the baser or animal faculties lie. The politician has an evenly rounded head, indicating a man whose chief desire is to use his fellow man, and when of no further use to advance his interests to throw him off as a child a toy. The murderer is wide between the ears, with little frontal development; the animal faculties being largely developed in the back and middle part of the skull, and dominating all others. These are the rough outlines of phrenology, and, if they hold good, why should not those of less prominence underlying others be patent to the touch of a skilled phrenologist? The face in physiognomy indicates the traits of character either by its coarse outlines or refined looks. If a clean-cut mouth, nostrils and a well defined chin are indicative, and they are always so, of an intelligent, sensitive, refined temperament, why should not the conformation of the head convey to one's mind a similar impression? All large heads have expansive, liberal thoughts, far-fetching and generous, while little heads, although bright on some subjects, are inclined as a rule to be narrow-minded. You can't make a pointer out of a bulldog, nor a race-horse out of a donkey. Each and all occupy their respective places. Then why try to qualify a brain for some position in life incapable of being taken. Some brains are suited for one thing, some for another. Where there is no artistic taste, how make a painter; where there is no faculty, could you drill a hole with a two-inch auger into that head and make an orator? They are graduated from the eminent divine to the politician, atheist and murderer.

The phrenologist upon examining the respective faculties as they are manifest by bumps on the skull will so inform the parents, and start the child to digging ditches, breaking rocks, carpentry, mechanics, or one of the professions, or some other vocation suited to his brain-power. A school established to teach phrenology, where young women could be educated in this science and give bent to the child's natural inclination would render a service of greater value to the community than the teaching of Latin or Greek to children incapable of comprehending the dead languages now taught in the public schools. The school would open an avenue to many competent, intelligent young women who plod and work with the needle day after day for the simple meal sufficient to maintain life. Women would be more suitable than men, for their naturally gentle, sensitive touch would more readily determine the protuberances on the skull than the calloused hand of the average man.

---

### DRAKE'S SUICIDAL MEAL.

It is alleged that Mrs. Robert Drake, of Covington, caused the death of her husband by the administration of sulphate of zinc and antimony. The case has attracted considerable attention. The defense furnished evidence to show that the dead man's last meal consisted of three slices of fresh pork, each five inches wide, seven inches long and a quarter of an inch thick. Over this he poured a quarter of a cup of vinegar from pickled onions. He also ate seven pickled onions, each about the size of a small walnut. He also ate a large slice of bread, two slices of warm bread, each slice ten inches long, five inches wide and half an inch thick, covered with butter. Drake also drank almost two cups of coffee and a quart of ginger tea. The defense will undertake to show that he was then seized with cholera morbus.

One witness testified that he had seen Drake eat twelve chicken legs, drink seven to eight cups of coffee at a sitting. "He had a wonderful appetite," said the witness. He had seen him drink patent medicine from the bottle, taking almost the entire contents at a dose.

By the memories of Jules Verne, and the shades of Baron Munchausen, what a gormandizer! What a capacity! Strange that the two emetics did not defeat the death angel.—*Med. and Surg. Monitor.*

